# Distribution analysis of gynecological carcinomas with concurrent second primary carcinomas

**DOI:** 10.3389/fonc.2026.1742872

**Published:** 2026-06-23

**Authors:** Si-yi Li, Chen-ying Liu, Xing-yun Xie, Lan-lan Chen, Wen-juan Chen

**Affiliations:** Department of Radiotherapy, Gynecology, Clinical Oncology School of Fujian Medical University, Fujian Cancer Hospital, Fuzhou, Fujian, China

**Keywords:** cervical carcinoma, distribution analysis, endometrial carcinoma, retrospective study, risk stratification, second primary carcinoma

## Abstract

**Introduction:**

This study investigates the distribution characteristics, occurrence time and clinicopathological correlations of second primary carcinoma (SPC) in patients with cervical carcinoma (CC) and endometrial carcinoma (EC) to guide precise monitoring.

**Materials and methods:**

This retrospective study analyzed 253 CC patients and 102 EC patients diagnosed with SPC at Fujian Cancer Hospital between April 2014 and April 2024. Descriptive statistical methods were employed to examine patient age, latency period, and clinicopathological characteristics. Additionally, a spatial distribution analysis of the anatomical sites of second primary cancers was conducted and visualized.

**Results:**

The median age at the initial diagnosis of CC and EC was 52 and 55 years, respectively. The median age of the SPC in CC and EC was 56 and 57.5 years; The median latency periods of SPC was 2 and 0.5 years. Among the CC patients who developed a SPC, 87.0% had cervical squamous cell carcinoma as the pathological type, and mainly in stages I-II(77.5%). The most common sites of SPC occurrence in CC were lung(25.3%), thyroid(22.5%), and breast(11.1%), and 69.1% are at stages I-II. Among the EC patients who developed a SPC, 87.3% have endometrioid adenocarcinoma as the pathological type, and 79.4% have been diagnosed at stage I-II. The SPC of EC mainly occurs in the thyroid(20.6%), ovary(16.7%), cervix(15.7%), and 82.4% occurs at stage I-II.

**Conclusion:**

This investigation on the occurrence of SPC following CC and EC uncovers distinct patterns in their locations and stages, profoundly influenced by their respective pathological types. These findings provide crucial insights for the re-examination of CC and EC, enabling earlier and more precise detection of SPC.

## 1 Introduction

Gynecological malignant tumors mainly include cervical carcinoma(CC), endometrial carcinoma(EC) and ovarian cancer (1), and they constitute an important part of the incidence and mortality rates among women worldwide. The second primary carcinoma (SPC) refers to cancers that occur simultaneously or successively in the same patient, where the patient has previously been diagnosed with one primary cancer, and SPC has been confirmed by pathology to not be a metastasis of the first primary cancer (2). With the improvement of medical awareness, the continuous advancement of diagnosis and treatment levels, and the increase in the accuracy of imaging scans, the overall survival period of patients with gynecological tumors has gradually increased.

Although HPV vaccination can effectively prevent the occurrence of CC (3), it still ranks among the top in the incidence and mortality rates of primary cancers in regions with low or medium human development indices. Moreover, as people’s living quality and economic level improve, the cumulative risk of cancer also increases. Gynecological tumors remain a major threat to women’s health worldwide (4). Currently, overweight and obesity have also been proven to be risk factors for increased occurrence of SPC in carcinoma patients (5). Recent studies have shown that the risk of SPC in gynecological tumor patients is significantly higher than that in the general population, and once SPC occurs, the prognosis is worse than that of patients with a single primary cancer (6). Therefore, the occurrence of SPC has gradually become an important issue affecting the long-term survival of gynecological tumor patients.

Currently, research on SPC in gynecological tumor patients is mostly focused on single cancer types, with relatively insufficient systematic epidemiological analysis (7) (8). Therefore, this study is based on clinical data from our hospital to comprehensively evaluate the occurrence characteristics of SPC in cervical cancer and endometrial cancer. The aim is to explore the role characteristics of pathological types and stages in it, in order to provide a basis for clinical formulation of long-term follow-up and individualized monitoring strategies.

## 2 Materials and methods

### 2.1 Data source and study subjects

This retrospective study enrolled patients with cervical cancer (CC) or endometrial cancer (EC) treated at Fujian Cancer Hospital between April 2014 and April 2024 who subsequently developed second primary cancers (SPCs). Clinical and pathological data were retrospectively collected. The study was approved by the Institutional Ethics Committee of Fujian Tumor Hospital ((Approval Date: 20250219; No. K2025-069-01). The final cohort consisted of 355 patients, including 253 with CC and 102 with EC.

### 2.2 Research methods

All first primary gynecological cancers included in this study were histologically confirmed. SPCs were diagnosed based on pathological confirmation of malignancy and comprehensive clinical evaluation. Recurrence or metastasis from the initial gynecological cancer was excluded through review of pathological findings, imaging studies, clinical course, and follow-up records.

Clinical data collected included age at diagnosis, histological subtype, disease stage, SPC site, latency, and survival outcomes. Categories represented by fewer than five cases were combined as “ other.” The category “lymph nodes or lymphoid tissue” refers to primary lymphoma and does not represent metastatic involvement of lymph nodes by solid tumors. Latency was defined as the interval between the diagnoses of the first primary cancer and SPC. SPCs diagnosed within 6 months were classified as synchronous, whereas those diagnosed after 6 months were classified as metachronous.

### 2.3 Statistical analysis

Statistical analysis employs R Studio (version 4.5.0) for code editing, generating corresponding graphs and analytical results. Quantitative data are presented as the median (interquartile range), while count data are described using frequency (percentage). Scatter-density combined graphs visually represent the distribution and temporal trends of data. This study utilizes these graphs to illustrate the age distribution and quantity changes among confirmed cases of CC and EC, as well as their SPC samples. The Sankey diagram effectively illustrates the direction and volume of flows between nodes—these flows constitute the core findings (9). Energy can transfer across different nodes; as depicted in our legend, this method clearly demonstrates the energy flow between various nodes. We apply the Sankey diagram to stratify SPC risk based on tumor histological type and the stage of the first primary cancer, and perform a descriptive analysis comparing survival between CC and EC patients.

## 3 Results

### 3.1 The difference of age in the diagnosis of primary cancer and SPC

**Figure 1:**
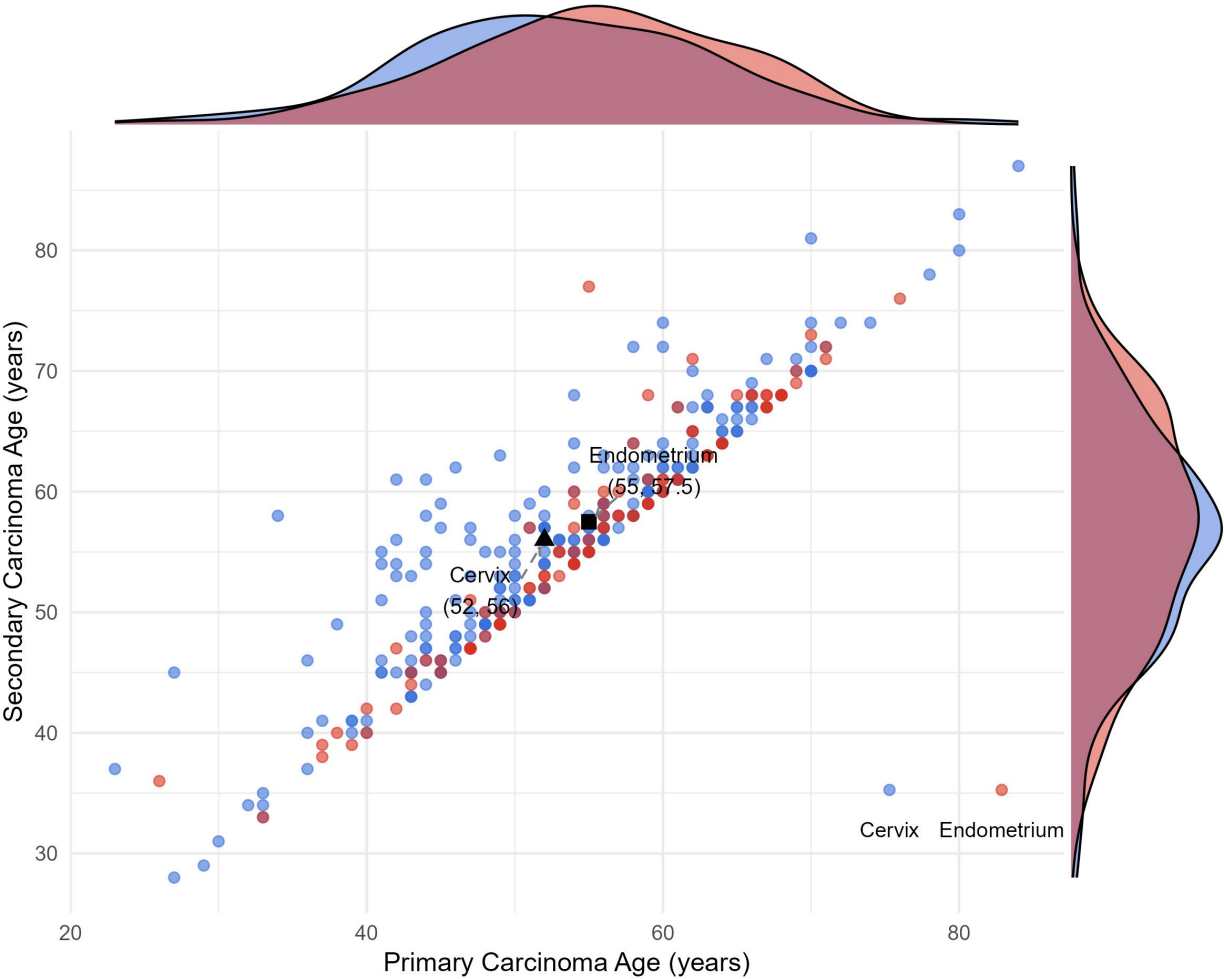
The difference of age in the diagnosis of primary cancer and SPC. **(A)** The pathological type of the first primary cancer; **(B)** the staging of the primary cancer; **(C)** the stage of the second primary cancer; **(D)** the location of the second primary cancer.

The median age at the first diagnosis of CC was 52 years (IQR: 46 - 60; 95% CI: 51 - 55), the median age at SPC diagnosis was 56 years (IQR: 50 - 62; 95% CI: 55 - 57), and the median latency period was 2 years (IQR: 0 - 4; 95% CI: 1 - 2). The median age at the first diagnosis of EC was 55 years (IQR: 49 - 62; 95% CI: 54 - 58), the median age at SPC diagnosis was 57.5 years (IQR: 49.25 - 64; 95% CI: 55 - 59.5), and the median latency period was 0.5 years (IQR: 0 - 2; 95% CI: 0 - 1). Changes in the age at the diagnosis of the first primary cancer and the corresponding second primary cancer are shown in **Figure 1**. **Table 1** presents the overall population distribution of second carcinoma in patients with cervical carcinoma. **Table 2** presents the overall population distribution of second carcinoma in patients with endometrial carcinoma.

**Table 1:** The population distribution of second carcinoma in patients with cervical carcinoma.

Second carcinoma site	Population with cervical carcinoma
Breast	28
Colon	10
Esophagus	6
Liver	7
Lung	64
Lymph nodes or lymphoid tissues	14
Other	15
Ovary	6
Rectum	8
Stomach	8
Thyroid	57
Uterus	19
Vagina	11

**Table 2:** The population distribution of second carcinoma in patients with endometrial carcinoma.

Second carcinoma site	Population with endometrial carcinoma
Breast	7
Cervix	16
Colon	7
Lung	14
Other	15
Ovary	17
Thyroid	21
Vagina	5

### 3.2 The diagnosis and staging distribution of cervical squamous cell carcinoma and SPC

**Figure 2:**
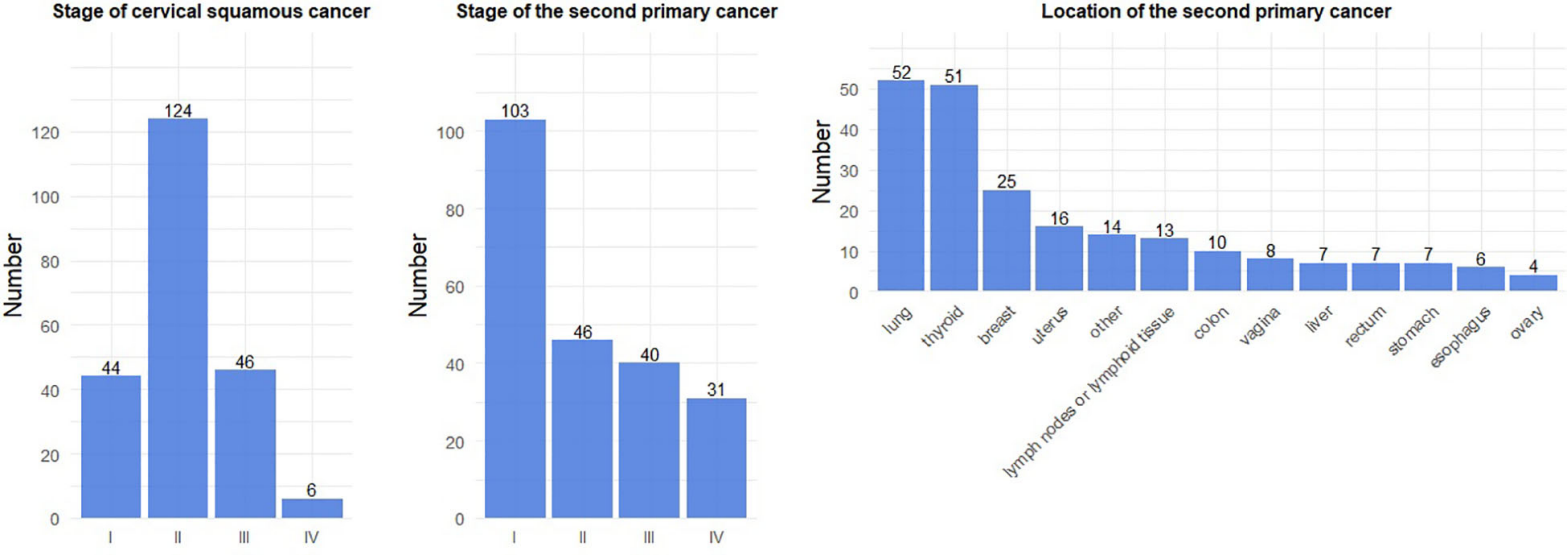
Distribution of CSCC stage and SPC stage & anatomic site.

**Figure 2** illustrates the distribution of cervical squamous cell carcinoma (CSCC) stages at diagnosis, along with the anatomical sites and stages of SPC. Among patients with CSCC, stage II disease was the most common at initial diagnosis (56.5%), followed by stage I (20.9%) and stage III (19.0%). Across all CSCC stages, most SPCs were diagnosed at stage I, whereas advanced-stage SPCs were less frequent. Lung and thyroid cancers were the most commonly observed SPCs, although the distribution varied according to the initial CSCC stage.

**Figure 3:**
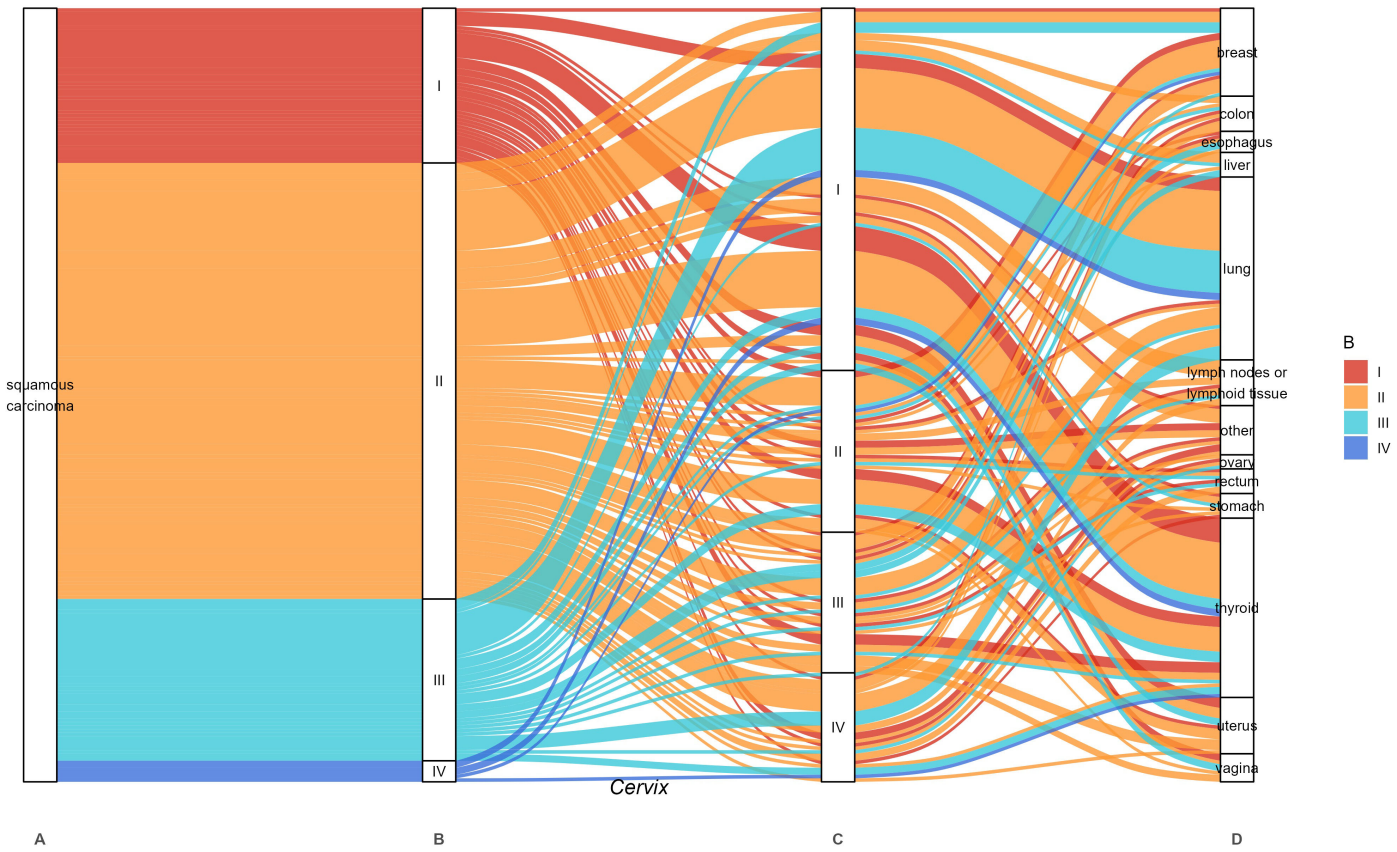
The relationship between the staging of CSCC and the location and stage of SPC. **(A)** The pathological type of the first primary cancer; **(B)** the staging of the primary cancer; **(C)** the stage of the second primary cancer; **(D)** the location of the second primary cancer.

**Figure 3** presents the distribution of SPC stages and sites according to CSCC stage. Across all CSCC stages, stage I SPC was the most common. Lung and thyroid cancers were the predominant SPC types. Stage II and III SPCs were less frequent and showed a more heterogeneous distribution, although thyroid, breast, and lung cancers remained among the most commonly affected sites. Stage IV SPC represented the smallest proportion of cases and was mainly observed in the lung, with no consistent site-specific pattern. Among patients with stage IV CSCC, the number of SPC cases was limited, precluding identification of a clear distribution pattern. Overall, early-stage SPC predominated regardless of CSCC stage, while lung and thyroid cancers were the most frequent SPC sites.

### 3.3 The diagnosis and staging distribution of cervical adenocarcinoma or adenosquamous carcinoma and SPC

**Figure 4:**
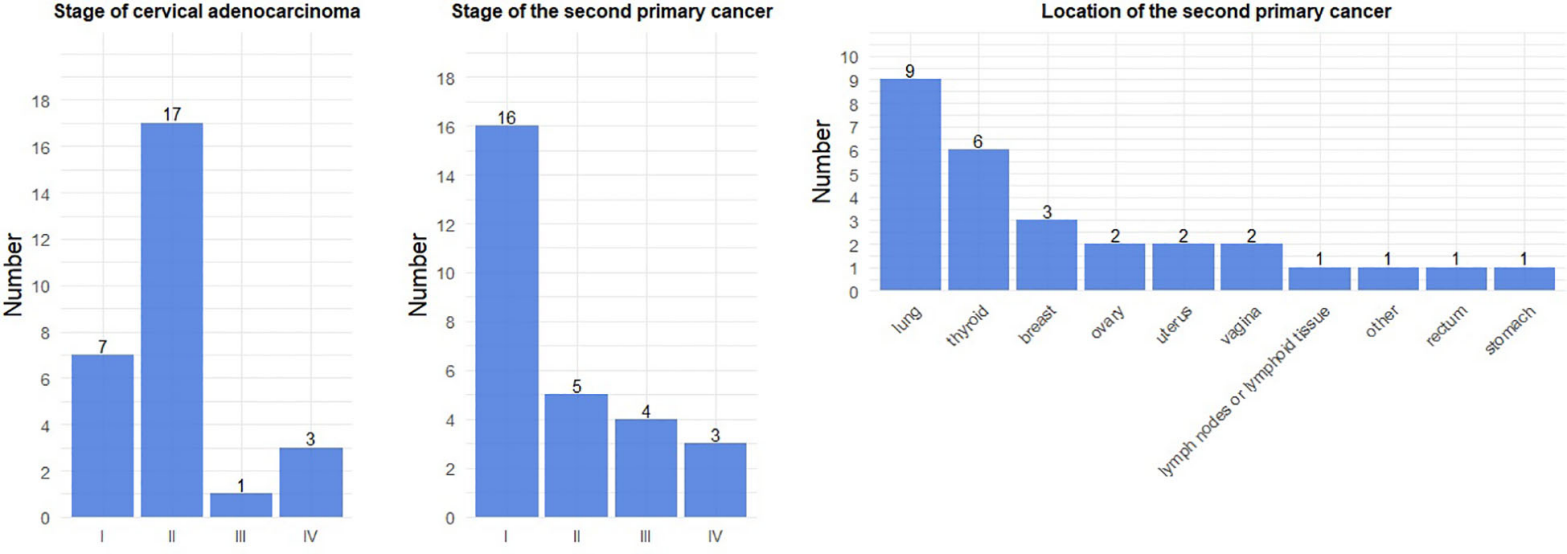
Distribution of cervical adenocarcinoma stage and SPC stage & anatomic site.

**Figure 5:**
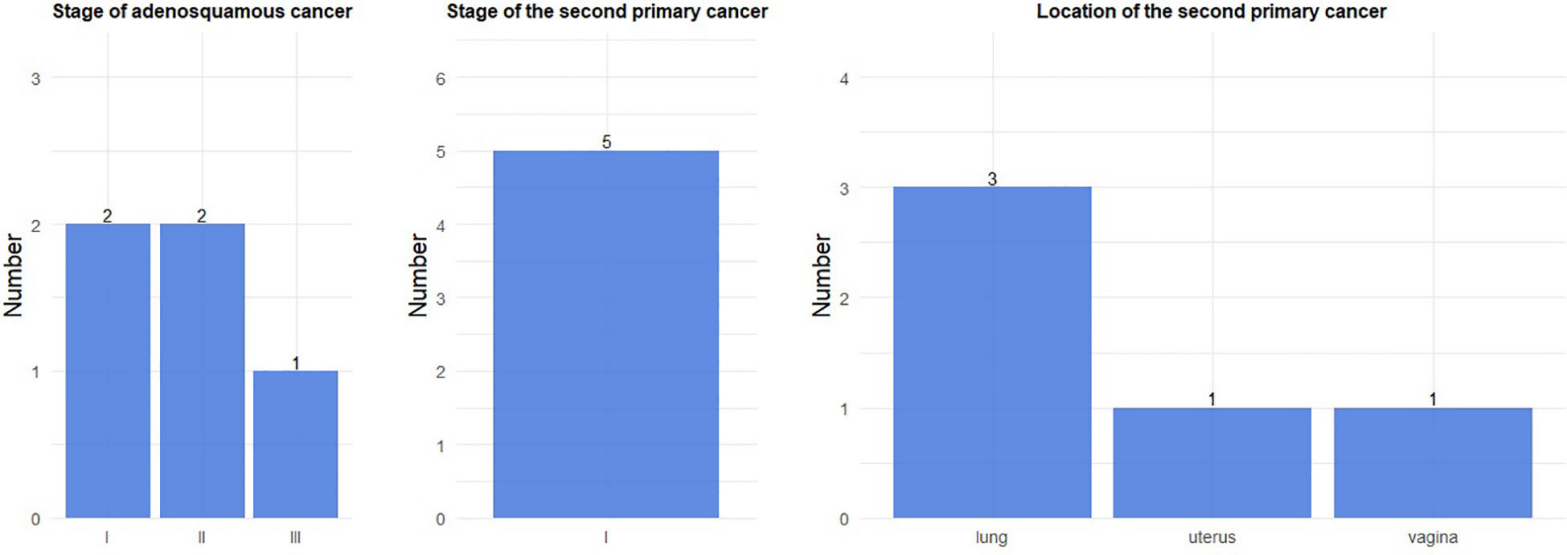
Distribution of cervical adenosquamous carcinoma stage and SPC stage & anatomic site.

**Figures 4**, **5** illustrates the distribution of clinical stages at diagnosis in patients with cervical adenocarcinoma and adenosquamous carcinoma, as well as the sites and stages of their SPC. Among patients with cervical adenocarcinoma, stage II was the most common at diagnosis (60.7%, 17/28), followed by stage I (25.0%, 7/28), stage IV (10.7%, 3/28), and stage III (3.6%, 1/28).

**Figure 6:**
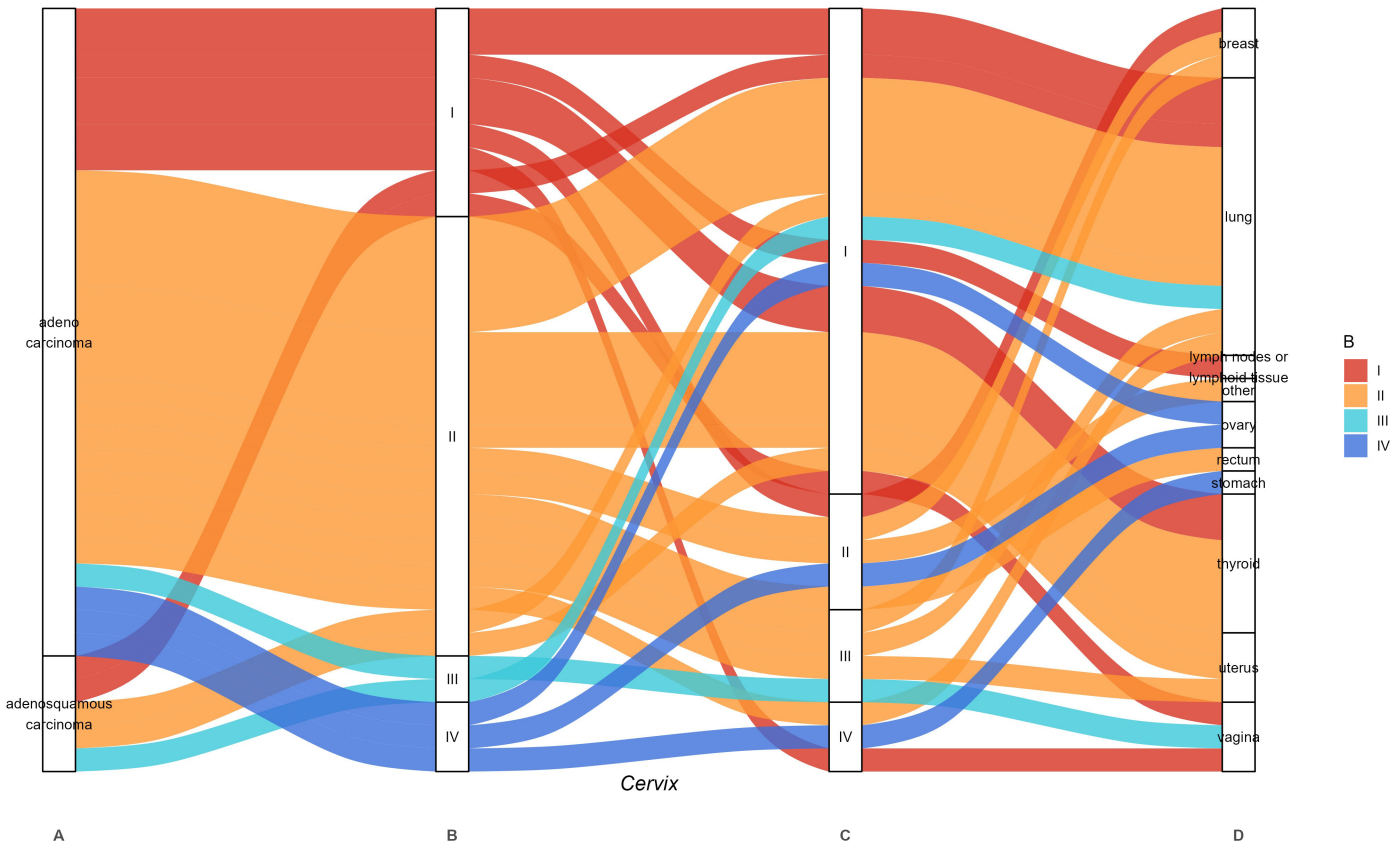
The relationship between the staging of cervical adenocarcinoma and adenosquamous carcinoma and the location and stage of SPC. **(A)** The pathological type of the first primary cancer; **(B)** the staging of the primary cancer; **(C)** the stage of the second primary cancer; **(D)** the location of the second primary cancer.

**Figure 6** summarizes the distribution of SPC stages and sites according to the stage of cervical adenocarcinoma and adenosquamous carcinoma. In patients with cervical adenocarcinoma, stage I SPC was the predominant subtype across disease stages, accounting for the majority of SPC cases. The lung and thyroid were the most frequent SPC sites, particularly among patients with stage I–II adenocarcinoma. Stage II and III SPCs were relatively uncommon and distributed across several organs, including the breast, uterus, rectum, and tongue. Advanced-stage adenocarcinoma (stages III–IV) was represented by only a few cases, and no consistent SPC pattern was observed.

Among patients with cervical adenosquamous carcinoma (n=5), stages at diagnosis included stage I (n=2), stage II (n=2), and stage III (n=1). All SPCs in this subgroup were diagnosed at stage I. The lung was the most frequently involved SPC site, whereas ovarian and uterine SPCs were observed only in isolated cases. Owing to the limited number of patients, no definitive stage-specific distribution pattern could be identified.

### 3.4 The diagnosis and staging distribution of endometrioid carcinoma and SPC

**Figure 7:**
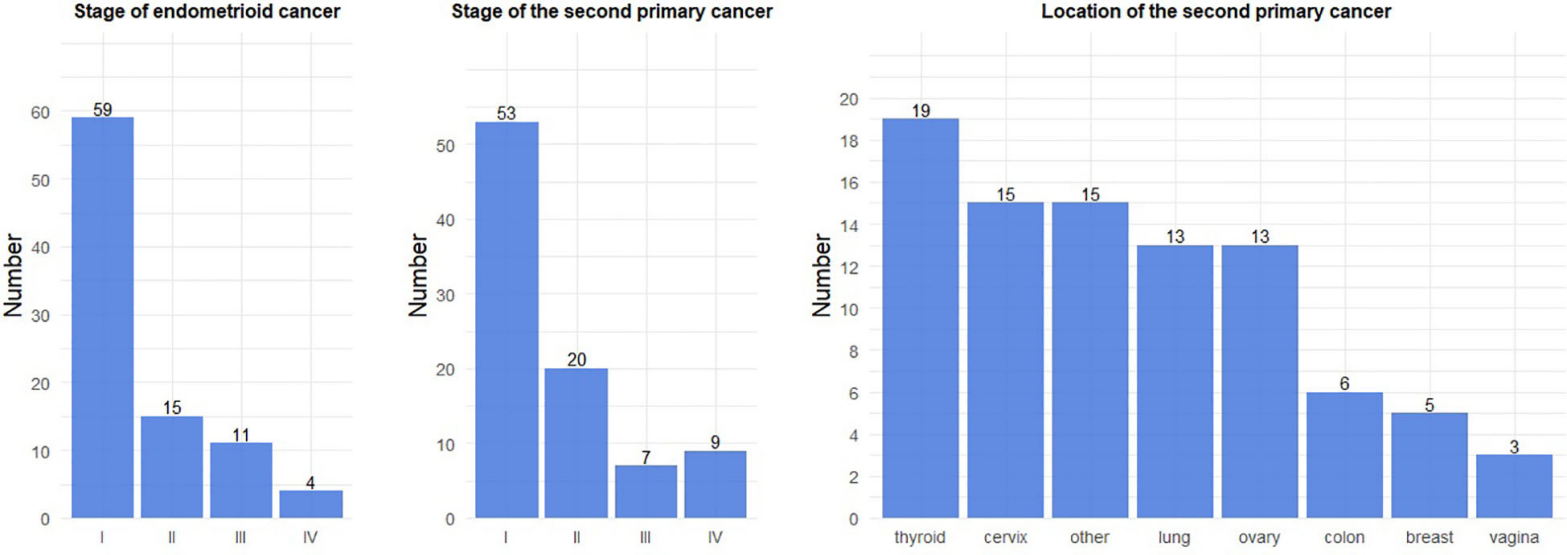
Distribution of cervical endometrioid carcinoma stage and SPC stage & anatomic site.

**Figure 7** summarizes the staging distribution of endometrioid carcinoma at diagnosis and the corresponding characteristics of SPC. At initial diagnosis, 59 patients (66.3%) presented with stage I EC, 15 (16.9%) with stage II, 11 (12.4%) with stage III, and 4 (4.5%) with stage IV.

**Figure 8:**
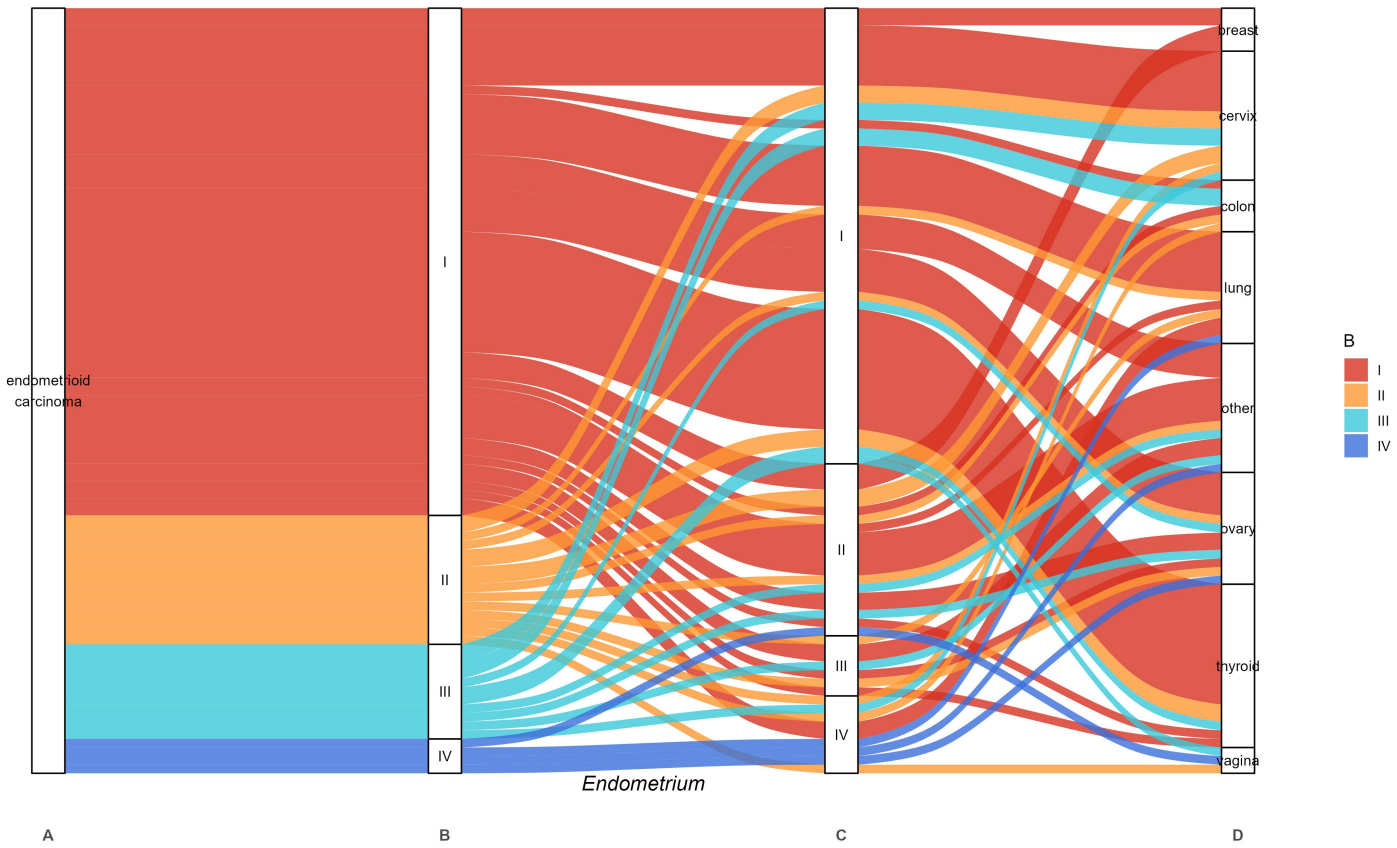
The relationship between the staging of endometrioid carcinoma and the location and stage of SPC. **(A)** The pathological type of the first primary cancer; **(B)** the staging of the primary cancer; **(C)** the stage of the second primary cancer; **(D)** the location of the second primary cancer.

**Figure 8** illustrates the distribution of SPC stages and sites according to the stage of endometrioid carcinoma. In patients with stage I endometrioid carcinoma, SPCs most commonly occurred in the thyroid, lung, cervix and ovary, with stage I SPC being the predominant presentation. In contrast, the cervix was the most frequent SPC site among patients with stage II–III endometrioid carcinoma. SPCs in patients with stage IV endometrioid carcinoma were infrequent and showed no distinct distribution pattern.

### 3.5 The diagnosis and staging distribution of endometrial mixed type or other type carcinoma and SPC

**Figure 9:**
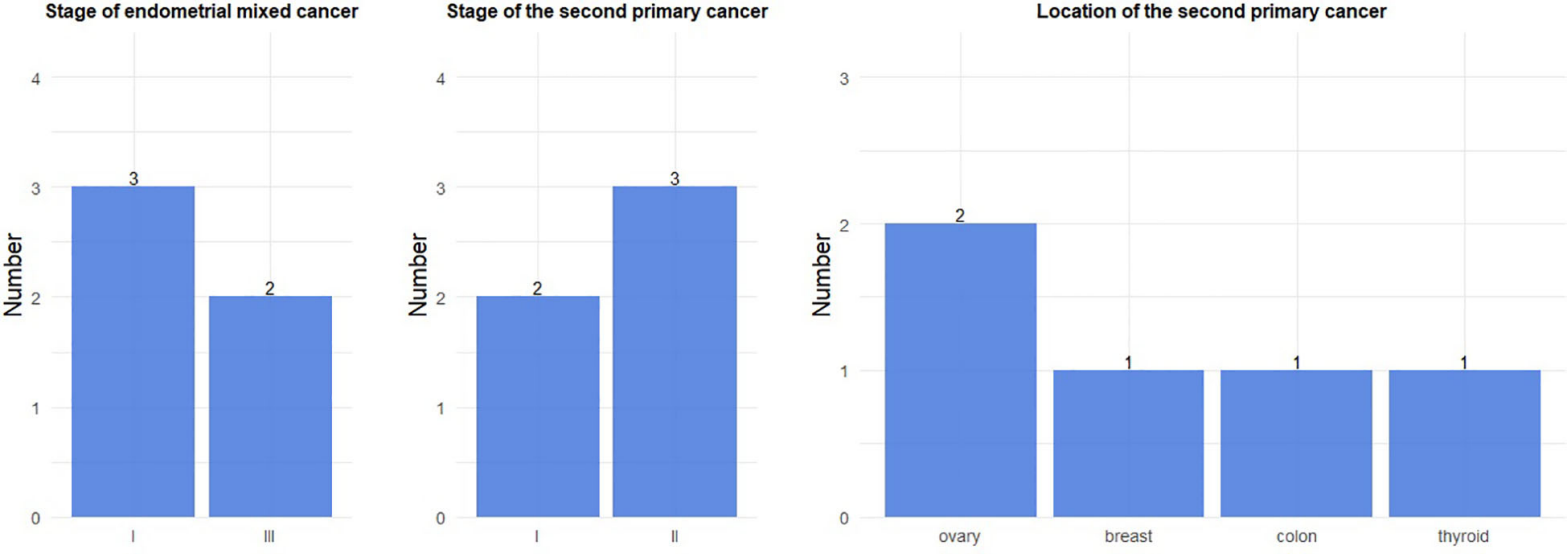
Distribution of endometrial mixed carcinoma stage and SPC stage & anatomic site.

**Figure 10:**
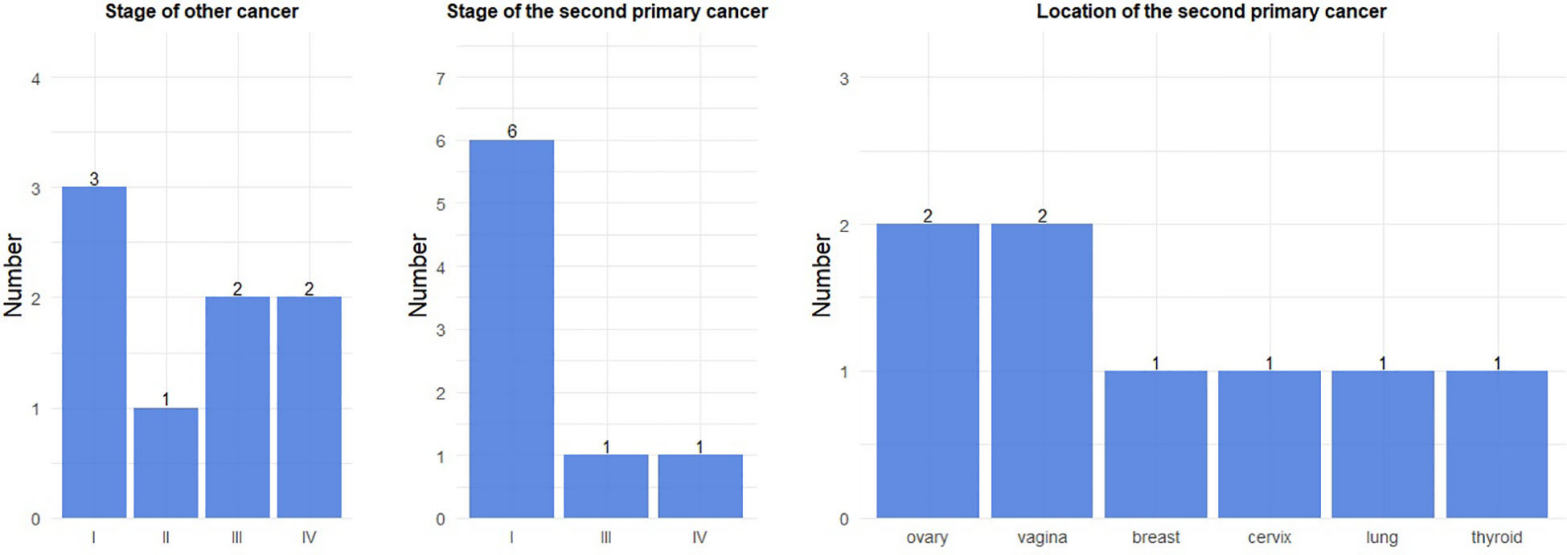
Distribution of endometrial other carcinoma stage and SPC stage & anatomic site.

**Figure 11:**
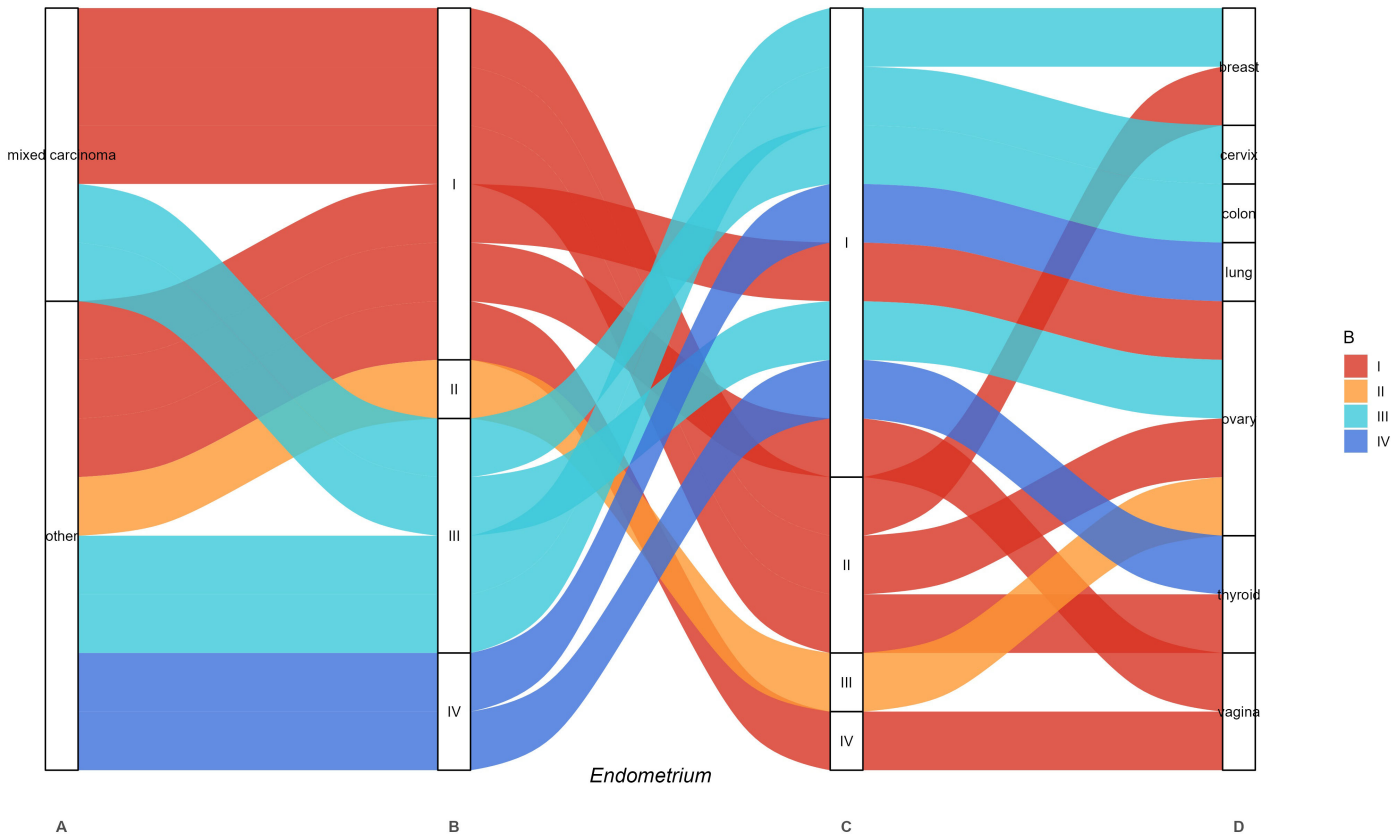
The relationship between the staging of endometrial cancer mixed carcinoma and other pathological types and the location and stage of SPC. **(A)** The pathological type of the first primary cancer; **(B)** the staging of the primary cancer; **(C)** the stage of the second primary cancer; **(D)** the location of the second primary cancer.

**Figures 9**–**11** the distribution of clinical stages at diagnosis in patients with endometrial mixed type or other type carcinoma, as well as the sites and stages of their SPC. Among patients with mixed carcinoma (n=5), three were diagnosed at stage I and two at stage III. SPCs were predominantly early stage. The ovary was the most common SPC site, while additional SPCs occurred in the thyroid, breast, and colon.

For other histological types, primary tumors were observed across all clinical stages. SPCs were predominantly stage I and showed a relatively scattered site distribution, with the ovary and vagina representing the most common sites, followed by the breast, cervix, lung, and thyroid. however, no clear association between primary tumor stage and SPC characteristics could be established because of the limited number of cases.

## 4 Discussion

Although cervical cancer and endometrial cancer are very common among female malignant tumors, the number of patients with SPC remains relatively small. This study, based on real-world data from Asian populations, is the first to systematically reveal the epidemiological characteristics of SPC in patients with CC and EC. The results show that the median latency period of SPC in cervical cancer patients is approximately 2 years, and that in endometrial cancer patients is approximately 0.5 years, suggesting that follow-up and screening should be strengthened within 2 years after the first diagnosis.

According to current research, SPC in patients with gynecological tumors predominantly manifests after the age of 50, with diagnoses occurring primarily at an early tumor stage. The characteristics of SPC are closely tied to the specific type of gynecological tumor. Notably, different pathological subtypes within the same tumor category may exhibit similar organ clustering patterns when concurrent with SPC. For instance, cervical cancer patients show a heightened incidence of SPC in the lungs, thyroid, and breast, while endometrial cancer patients are more prone to developing gynecology-related second primary cancers.

Our analysis of CC revealed distinct, stage-dependent patterns in the occurrence and distribution of SPC among patients with CSCC. Across all stages, the lung, thyroid, and breast consistently emerged as the most common sites for SPC, with the lung predominating in advanced CSCC (stages II–III) and the thyroid appearing more frequently in early-stage disease. Notably, more than half of the SPC cases in stage I–II CSCC were diagnosed at an early stage, suggesting a potential for timely detection through vigilant follow-up. In contrast, patients with stage III–IV CSCC more often developed SPC at advanced stages, particularly within the lung, gastrointestinal tract, and liver. Similarly, SPC in cervical adenocarcinoma and adenosquamous carcinoma showed preferential involvement of the lung, thyroid, and breast, with a clear predominance of early-stage lesions.

For early-stage EC(stage I), the development of SPC most frequently involved the thyroid, lung, and ovary. These SPCs were predominantly diagnosed at an early stage, suggesting a possible link to endocrine-related mechanisms or an underlying genetic susceptibility. In contrast, EC diagnosed at stages II–III was more frequently associated with SPCs of the cervix and gastrointestinal tract, which tended to be detected at a later stage. This pattern may reflect shared carcinogenic environments within the pelvis or influences related to prior treatment.

Those phenomenon is mainly attributed to the following factors: ① Human papillomavirus (HPV) plays a crucial role in the onset of cervical cancer (10). In recent years, several studies have shown that HPV infection may also be a potential risk factor for thyroid cancer (11). Further research can explore whether HPV infection is an independent risk factor for concurrent thyroid cancer in cervical cancer. ② Smoking is the main risk factor for lung cancer, which is a consensus (12). At the same time, smoking is also a clear risk factor for cervical cancer (13). ③ As the most common endocrine system cancer, thyroid cancer interferes with crucial bodily functions controlled by the thyroid. The incidence of cervical cancer patients can increase with age and the increase of FSH (14). It may affect the release of thyroid-stimulating hormone by the hypothalamus-pituitary-gonadal axis, leading to thyroid dysfunction and affecting the occurrence of thyroid cancer. ④ Sex hormone imbalance plays a key role in the occurrence of endometrial cancer and ovarian cancer. ⑤ Lynch syndrome is the only known hereditary syndrome of endometrial cancer (15). It can affect the endometrium and ovaries, etc., and it increases the risk of synchronous or metachronous tumors in the endometrium and ovaries.

This study focused on patients with double primary gynecological tumors, finding they were predominantly synchronous cancers, primarily identified through pathological diagnosis—the gold standard for tumor confirmation (16). The main symptoms mirrored those of single gynecological tumors; notably, when the second primary lesion was too small, it risked misdiagnosis or oversight during initial clinical assessment. Currently, no effective indicators exist to promptly detect such cases. Therefore, based on this study’s conclusions, heightened emphasis should be placed on screening for second primary cancers across the female reproductive organs in endometrial cancer patients, aiming to facilitate timely detection of a concurrent primary malignancy.

Key strengths of this study include the relatively large cohort size, the focus on an Asian population, the comprehensive evaluation of clinicopathological characteristics associated with SPCs, and the use of Sankey diagrams to visualize SPC patterns. Moreover, the identification of a short latency period (0–2 years) and distinct SPC distribution patterns between cervical and endometrial cancer patients provides clinically relevant evidence for risk-adapted surveillance strategies.

This study is limited by its retrospective design, which may introduce selection bias. External validation across multiple centers is lacking. The absence of a control cohort without secondary primary cancers hinders identification of independent risk factors. Detailed comparisons between synchronous and metachronous cases were not performed. Moreover, molecular and genetic data were unavailable, and treatment-related factors were not systematically assessed. Future studies should incorporate comprehensive treatment stratification to better evaluate therapeutic impacts.

## 5 Conclusion

In summary, SPC in patients with cervical cancer are commonly found in the lungs, thyroid, and breasts, while SPC in patients with endometrial cancer are more frequently observed in the thyroid, ovaries, and cervix, and are also mostly early-stage cases. The findings of the present study suggest that intensified surveillance of anatomical sites at high risk for SPC occurrence and sustained follow-up of the primary disease during the first two years after diagnosis may help improve outcomes in patients with CCand EC.

## Data Availability

The raw data supporting the conclusions of this article will be made available by the authors, without undue reservation.
